# Role for the Histone Demethylase KDM4B in Rhabdomyosarcoma via CDK6 and CCNA2: Compensation by KDM4A and Apoptotic Response of Targeting Both KDM4B and KDM4A

**DOI:** 10.3390/cancers13071734

**Published:** 2021-04-06

**Authors:** Zoë S. Walters, Ewa Aladowicz, Barbara Villarejo-Balcells, Gary Nugent, Joanna L. Selfe, Paul Eve, Julian Blagg, Olivia Rossanese, Janet Shipley

**Affiliations:** 1 Divisions of Molecular Pathology and Cancer Therapeutics, The Institute of Cancer Research, Sutton, London SM2 5NG, UK; z.s.walters@soton.ac.uk (Z.S.W.); ewa.aladowicz@icr.ac.uk (E.A.); Barbara.Villarejo@icr.ac.uk (B.V.-B.); Joanna.Selfe@icr.ac.uk (J.L.S.); 2 Cancer Sciences, Faculty of Medicine, Southampton General Hospital, Southampton SO16 6YD, UK; 3 Division of Cancer Therapeutics, The Institute of Cancer Research, Sutton, London SM2 5NG, UK; Gary.nugent@benevolent.ai (G.N.); Paul.Eve@icr.ac.uk (P.E.); julian.blagg@neophore.com (J.B.); olivia.rossanese@icr.ac.uk (O.R.)

**Keywords:** KDM4B, histone demethylases, rhabdomyosarcoma, epigenetics

## Abstract

**Simple Summary:**

Rhabdomyosarcomas (RMS) are soft tissue sarcomas predominant in the pediatric age range. Current standard multi-modal treatments can result in severe life-long side-effects across all subtypes and dismal outcomes in RMS patients classified as high-risk. There is an urgent need to find novel, more targeted therapies for these children. High expression of a number of histone demethylases is shown to support proliferation of RMS cells and here we direct our attention on KDM4B which has become a recent focus for drug discovery efforts. We show that KDM4B regulates cell cycle genes in the context of RMS cells and demonstrate that long-term silencing of KDM4B is functionally compensated for by another KDM4 family member, KDM4A. Upfront reduction of both KDM4A and KDM4B kills RMS cells demonstrating that therapeutics designed to target both histone demethylase family members is desirable to circumvent functional redundancy.

**Abstract:**

Histone demethylases are epigenetic modulators that play key roles in regulating gene expression related to many critical cellular functions and are emerging as promising therapeutic targets in a number of tumor types. We previously identified histone demethylase family members as overexpressed in the pediatric sarcoma, rhabdomyosarcoma. Here we show high sensitivity of rhabdomyosarcoma cells to a pan-histone demethylase inhibitor, JIB-04 and identify a key role for the histone demethylase KDM4B in rhabdomyosarcoma cell growth through an RNAi-screening approach. Decreasing KDM4B levels affected cell cycle progression and transcription of G1/S and G2/M checkpoint genes including *CDK6* and *CCNA2*, which are bound by KDM4B in their promoter regions. However, after sustained knockdown of KDM4B, rhabdomyosarcoma cell growth recovered. We show that this can be attributed to acquired molecular compensation via recruitment of KDM4A to the promoter regions of *CDK6* and *CCNA2* that are otherwise bound by KDM4B. Furthermore, upfront silencing of both KDM4B and KDM4A led to RMS cell apoptosis, not seen by reducing either alone. To circumvent compensation and elicit stronger therapeutic responses, our study supports targeting histone demethylase sub-family proteins through selective poly-pharmacology as a therapeutic approach.

## 1. Introduction

Epigenetic marks on chromatin and the enzymes that modulate them are emerging as key mechanisms underlying cancer tumorigenesis and progression. Histone modifications are central to controlling chromatin architecture, gene transcription and DNA repair, and a number of proteins controlling the reading, writing and erasing of such marks are aberrant in cancer with targeted epigenetic modulators against them undergoing clinical trials [1].

Histone demethylases (HDMs) are a family of epigenetic modulators responsible for the removal of methyl groups from specific residues on histone tails and have been shown to play a role in stem cell identity and differentiation [2,3]. The KDM4 family in particular has been linked to stem cell self-renewal and regulation of differentiation. Overexpression of KDM4A results in mammosphere formation in non-transformed breast cells, indicative of cancer stem cell formation, and silencing of KDM4 family members has been shown to induce differentiation in embryonic stem cells [4,5,6]. The apparent coincident role of the KDM4 family members in the maintenance of stem cell identity is suggestive of functional redundancy between these proteins although non-redundant and overlapping roles for KDM4 family members have been indicated [2,3].

KDM4 family members have also been shown to be genetically aberrant and overexpressed in certain malignancies [7,8]. Moreover, silencing of KDM4 family members has been shown to reduce proliferation and induce differentiation in a number of tumor types [9].

Rhabdomyosarcomas (RMS) are a type of soft tissue sarcoma that resemble developing skeletal muscle and are a major cause of cancer-related deaths in children and adolescents. Despite an overall increase in survival rate over the past few decades, drug resistance is a major factor in treatment failure [10]. A number of studies have highlighted therapeutically targetable proteins that regulate epigenetic changes and are modulators of RMS cell growth [11,12,13,14,15]. Previously we have shown that certain HDM family members are highly expressed in RMS [15]. We have also shown that JARID2, a non-catalytic HDM family member that is part of the polycomb repressive complex 2 (PRC2) is required for the undifferentiated state of RMS cells. Here we demonstrate sensitivity of RMS cell lines to the pan-jumonji demethylase inhibitor JIB-04 [16] that alters H3K9me3 status and show that the H3K9me3 demethylase KDM4B is highly expressed in RMS. Silencing of KDM4B led to a reduction in proliferation in cell lines representing both the embryonal and alveolar subtypes of RMS. We also show that KDM4B regulates the expression of cell cycle checkpoint control genes in RMS, including *CDK6* and *CCNA2* via H3K9me3 at their promoters. However, after sustained silencing of KDM4B, we also show that recovery of cell growth is attributable to KDM4A recruitment to the promoters of *CDK6* and *CCNA2*. Furthermore, upfront knockdown of both KDM4B and KDM4A led to cell apoptosis, not seen with silencing of either demethylase alone. Taken together, our results support therapeutic potential for RMS patients by KDM4 family inhibition.

## 2. Materials and Methods

### 2.1. Cell Culture

RMS cell lines RH30, RD, RMS-YM, RH4, JR-1, RMS-01, RH41, HFF-1 have been described previously [17]. Cell lines were cultured in Dulbecco’s modified Eagle’s medium (Thermo Fisher Scientific, Waltham, MA, USA) (RD, RH30, RMS-01, RH4) or Roswell Park Memorial Institute 1640 (RPMI) medium (Thermo Fisher Scientific, Waltham, MA, USA)(RH41, RMS-YM) supplemented with 10% fetal calf serum (Gibco, Life Technologies Ltd., Thermo Fisher Scientific, Waltham, MA, USA), 2 mM L-glutamine (Thermo Fisher Scientific, Waltham, MA, USA) and 1% penicillin/streptomycin (Thermo Fisher Scientific, Waltham, MA, USA). HFF-1 cells were purchased from ATCC and cultured in Dulbecco’s modified Eagle’s medium supplemented with 15% fetal calf serum, 2 mM L-glutamine and 1% penicillin/streptomycin. Cells were maintained at 37 °C at 5% CO_2_.

Cell lines were authenticated using the Geneprint 10 kit according to manufacturer’s instructions (Promega, Madison, WI, USA) and subsequent fragment length analysis was carried out by Eurofins Genomics (Ebersberg, Germany). The resulting short tandem repeat (STR) typing results were compared with public databases.

### 2.2. siRNA Screen and Transfections

**Figure 1:**
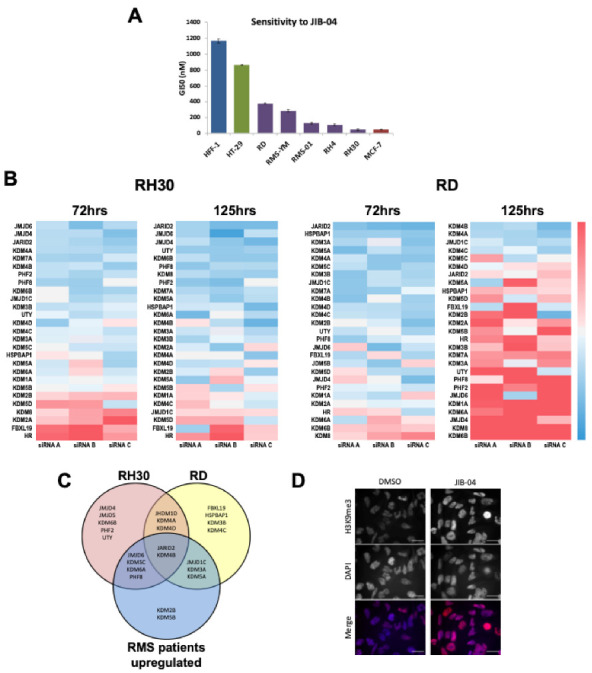
Histone Demethylase (HDM) family members are important in RMS cell growth. (**A**) GI50 sensitivity of RMS cell lines (purple) to JIB-04 compared with insensitive HFF-1 (blue) and HT-29 (green), and sensitive MCF-7 (red) cell lines. (**B**) Silencing of 27 known HDM family members in 2 RMS cell lines (RH30, ARMS fusion positive and RD, ERMS fusion negative) at 2 timepoints (72 h and 125 h) using 3 siRNAs per HDM. Heatmaps represent cell viability of each siRNA relative to normal control siRNA (Blue = reduction in proliferation, red = increase). (**C**) Venn diagram depicting HDMs with >2 siRNAs with a Z score of <−0.5 at both timepoints for each cell line (RH30 = red, RD = yellow) overlapped with HDMs with greater than 2-fold expression (ITCC/CIT dataset, [15,18]) in RMS patient samples relative to normal skeletal muscle (blue, [12]). (**D**) JIB-04 treatment (150 nM) results in an increase in H3K9me3 by immunofluorescence in RH30 cells, consistent with inhibition of KDM4 family members (scale bar = 10 um).

For siRNA silencing experiments 3 siRNAs were used for each histone methyltransferase family member. siRNAs were synthesised by Sigma and RMS cell lines were transfected using Lipofectamine RNAiMAX (Invitrogen, Carlsbad, CA, USA) with siRNAs at a final concentration of 10 nM. The data was subsequently processed and analysed using python 3 using pandas, numpy, matplotlib, seaborn libraries (Figure 1B). For subsequent KDM4B siRNA transfections for cell proliferation, cell cycle and qRT-PCR analysis siRNAs were transfected as described above. For dual knockdown experiments, siRNA mixtures of two individual siRNAs were made to a final concentration of 10 nM (5 nM per siRNA).

### 2.3. Drug Sensitivity Analysis

Cells for dose-response curves using were plated in 96-well plates and JIB-04 (Sigma-Aldrich, Gillingham, Dorset, UK) was added at increasing dose increments from 0 (DMSO (Sigma-Aldrich) only) to 10 µM and growth measured using using CellTiter 96^®^ AQueous One Solution Cell Proliferation Assay (MTS) kit (Promega) at 96 h. GI_50_s were calculated in GraphPad Prism 7 (GraphPad Software Inc., San Diego, CA, USA) using the mean of six replicates. 

### 2.4. Immunofluorescence

Cells were fixed in 2% paraformaldehyde in PBS, permeabilized in 0.1% Triton X-100, blocked with 10% normal donkey serum with 1% BSA, and incubated with primary antibody O/N. Alexa Fluor 555-conjugated secondary antibody was used. Nuclei were stained with DAPI before fluorescence imaging (Axioplan2, Carl Zeiss Microscopy, Oberkochen, Germany). Antibodies listed in Table S7. 

### 2.5. Immunohistochemistry

The immunohistochemistry was performed on tissue microarrays containing samples from 219 RMS patients as well as normal tissues. Tissue microarrays were deparaffinized, hydrated through graded alcohol series and rinsed in water. After antigen retrieval by HIER, peroxide blocking was performed with 3% H_2_O_2_ at room temperature for 5 min. The sections were blocked with 4% BSA and incubated with KDM4B antibody at 4 °C overnight. Then, the samples were probed with secondary Ab (ChemMate™ EnVision™/HRP, Rabbit/Mouse (ENV), K5007, DAKO, Denmark), followed by incubation with peroxidase substrate solution (DAB, DAKO, K5007) and counterstained with hematoxylin.

The immunoreactions were evaluated independently by two pathologists blinded to the clinicopathologic information. KDM4B immunostaining was evaluated based on the percentage of positively stained tumor cells. Additionally, the staining intensities were classified into the following four categories: 0 (no staining); 1 (weak staining); 2 (moderate staining), 3 (strong staining) in at least 10% of cells. 

### 2.6. KDM4B Gene Expression

Gene expression data described previously [15,18] was analysed for KDM4B expression. KDM4B expression in patient samples and cell lines was analysed by TaqMan (Life Technologies Ltd., Thermo Fisher Scientific, Waltham, MA, USA) after RNA extraction by Trizol (Thermo Fisher Scientific) according to the manufacturer’s instructions and cDNA synthesis using Superscript II (Thermo Fisher Scientific). 

### 2.7. Cell Proliferation, Cell Cycle and Apoptosis Assays

Cell proliferation assays were carried out using CellTiter 96^®^ AQueous One Solution Cell Proliferation Assay (MTS) kit (Promega) and CyQuant Proliferation Assay kits (Invitrogen, Carlsbad, CA, USA) according to the manufacturer’s instructions. For cell cycle analysis cells were harvested after siRNA silencing, washed in 1XPBS fixed in 70% ethanol and stained for 30 min with propidium iodide (Invitrogen). Cells were then analyzed for to cell cycle distribution on an LSR II (BD, Oxford, UK). Apoptosis assays were carried out after siRNA transfection using Caspase Glo assay (Promega). 

### 2.8. RNA-Sequencing and Bioinformatics

RNA for RNA-sequencing was extracted 48 and 72 h post-transfection with either a non-targeting control siRNA or one of 2 KDM4B siRNAs (two replicates of each). RNA was sent to Oxford Gene Technology (OGT, Oxford, UK) for library preparation and sequencing. Gene expression data was obtained and analysed for differential gene expression between control siRNAs and KDM4B siRNAs.

The diff files from the RNASeq were processed using R (version 3.1.4., R Foundation for Statistical Computing, Vienna, Austria) using dplyr, ggplot2, gplots, RColorBrewer packages to manipulate the data and generate the scatterplots and heatmaps. The full data set was clustered using the R hclust hierarchical clustering function. Using this the data was split into 10 clusters for uploading to genego.com for GO analysis. Further analysis of the clusters was analysed in Python 3.

### 2.9. Western Blotting

Protein expression of KDM4 family members and cell cycle proteins was carried out by Western blotting. Protein from transfected cells was extracted using cell lysis buffer (Cell Signaling Technology, Danvers, MA, USA). Proteins were resolved on 3–8% Bis-Tris acrylamide gels and transferred onto PVDF membranes using the X-Cell II and iBlot systems according to manufacturer’s instructions (Invitrogen). Antibodies can be found in Table S7.

### 2.10. Chromatin Immunoprecipitation and qPCR

Chromatin immunoprecipitations (ChIP) were carried out using the SimpleChIP Enzymatic Chromatin IP kit in accordance with the manufacturer’s instructions (New England Biolabs Inc, MA, USA). Eluted chromatin underwent qPCR analysis using primers specific to promoter regions of CDK6 and CCNA2. Primary antibodies can be found in Table S7.

### 2.11. shRNA Transfections

Stable KDM4B and control shRNAs were packaged into virus in HEK293T cells as described previously [17]. HEK293T cells were transfected at 50% confluence using jetPEI transfection reagent (Polyplus transfection, Illkirch, France). After 24 h culture media was replaced. Viral particles were collected at 48 and 72 h post-transfection. RMS cell lines were transduced with lentiviral particles at 90% confluence and subject to puromycin selection for 72 h. Surviving cells were re-plated and assessed for cell proliferation by MTS assay (Promega) at stated timepoints. Silencing of KDM4B was confirmed by western blotting at 144 h post-selection. 

### 2.12. Statistics

Each examination was performed in at least triplicate. For in vitro experiments, statistical differences were analyzed using Welch’s *t*-test (two-tailed) in GraphPad Prism 8.0.2 softwaree. Data is presented as the mean ± standard deviation. Significance is indicated as *p* < 0.05 (*), *p* < 0.01 (**) and *p* < 0.001 (***), while NS indicates a non-significant *p*-value.

## 3. Results

### 3.1. HDMs Are Important Mediators of Proliferation In Rhabdomyosarcomas

As we had previously shown that a number of demethylase gene family members are overexpressed in RMS relative to normal skeletal muscle samples [15] we determined the sensitivities of RMS cell lines to an established pan-jumonji demethylase (HDM) inhibitor, JIB-04 [16]. We treated RMS cell lines and the non-transformed normal fibroblast cell line HFF-1 with increasing concentrations of JIB-04 over a 96 h period to determine the GI_50_ and compared these with known sensitive (MCF-7) and insensitive (HT-29) cell lines [16]. All RMS cell lines were sensitive to JIB-04 (GI50 < 500 nM), whereas the non-tumorigenic HFF-1 cell line insensitive (Figure 1A).

To determine which HDMs are important for RMS cell growth and to further understand the sensitivity of RMS cell lines to JIB-04, we performed an siRNA screen in 2 RMS cell lines representative of the RMS subtypes, embryonal RMS (ERMS, RD cell line) and alveolar RMS (ARMS, RH30 cell line), at two time-points using three siRNAs for each HDM (Figure 1B, Figure S1A–F). Briefly, three siRNAs were designed to target each of 27 HDMs. Cell proliferation was measured at two time-points after transfection, 72 h and 125 h. Reduction in cell proliferation was calculated relative to a non-targeting control siRNA (Table S1) and HDMs were considered hits where at least two siRNAs gave a *z*-score of >−0.5 at either time point in each of the two cell lines. Overlaying the hits from the screen with those determined to be overexpressed in RMS patient samples, both fusion positive and negative (fold change >2, *p* < 0.0001, [13]) identified JARID2 which we have previously investigated [13] and the H3K9me3 HDM KDM4B and (Figure 1C). After treatment with the JIB-04 pan-HDM inhibitor we observed an increase in levels of the H3K9me3 mark (Figure 1D). Taken together with the siRNA data, this indicates that KDM4 demethylase activity contributes to the sensitivity of RMS cells to JIB-04. 

### 3.2. KDM4B Is Highly Expressed and Correlates with A Proliferative Marker in RMS

**Figure 2:**
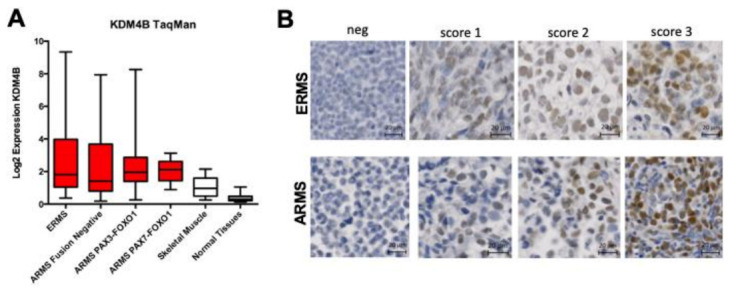
KDM4B is overexpressed in RMS patients. (**A**) KDM4B expression by RMS subtype relative to skeletal muscle and normal tissue expression. (**B**) Photomicrograph of tissue microarray cores, showing examples of KDM4B expression in ERMS and ARMS samples scored as 0, 1, 2 or 3 in intensity by an expert pathologist (scale bars = 20 μm).

KDM4B expression levels were analyzed in 125 RMS samples by qRT-PCR and results compared to normal skeletal muscle. We found that KDM4B is highly expressed in all RMS subtypes when compared to normal skeletal muscle (log2 Diff. in means = 1.417, *p* < 0.0001, one-way analysis of variance) and a panel of normal tissues (Log2 Diff. in means = 2.106, *p* < 0.0001, one-way analysis of variance), with no statistical difference in expression between fusion gene positive and negative RMS (Figure 2A, Figure S2C). These results are consistent with analyses of our previously published microarray data [18] where KDM4B is >2-fold overexpressed in all RMS patients at the mRNA level when compared to skeletal muscle [15] (Figure 1C, Figure S2A,B). 

KDM4B protein levels were also determined in 219 RMS patient samples by immunohistochemistry (IHC), all of which have long-term follow-up clinical information available. 40% of RMS were positive for KDM4B staining (>10% cells stained), comparing to negative staining in normal muscle (*p* < 0.001) (Figure 2B, Figure S3, Table S2), consistent with our analysis of qRT-PCR data. Expression levels of KDM4B did not correlate with clinical outcome data based on either histology or the presence of metastasis (Table S3). Based on previous reports of a role for KDM4B in cell proliferation (reviewed in [9]), we tested for a correlation between expression levels of KDM4B and Ki-67, a marker of cell proliferation. Among the 219 cases, analysis of the immunohistochemistry results indicated that KDM4B and Ki-67 protein levels were positively associated (Chi squared test for trend, *p* = 0.001) (Table S4), consistent with a role for KDM4B in RMS cell proliferation.

### 3.3. KDM4B Silencing Results in A Reduction in Proliferation in RMS cells 

**Figure 3:**
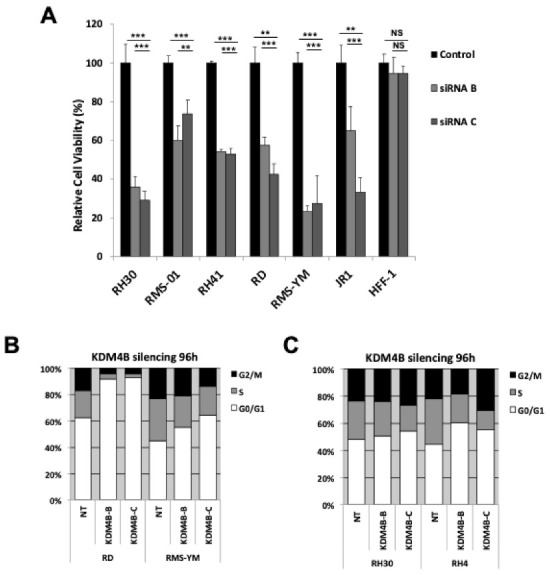
KDM4B reduction decreases RMS cell proliferation. (**A**) Silencing of KDM4B in multiple RMS cell lines and a normal control cell line, HFF-1. Numbers represent % of cells remaining at 96 h post-transfection relative to normal control siRNA. Results are presented as mean ± SD. Significance is indicated as *p* < 0.01 (**) and *p* < 0.001 (***), while NS indicates a non-significant *p*-value. Cell cycle analysis of KDM4B-silenced RMS cells; KDM4B silencing increases G1 arrest in ERMS cell lines (**B**) KDM4B silencing results in a reduction in cell cycle progression in ARMS cell lines (**C**).

A role for KDM4B in cell proliferation was assessed after RNAi-mediated silencing of KDM4B using KDM4B-specific siRNAs in 6 RMS cell lines representing both ERMS and ARMS and compared with HFF-1 cells. KDM4B silencing resulted in a reduction in cell proliferation in both ARMS and ERMS cell lines of up to 70% by 96 h post transfection (Figure 3A, Figure S4A,B). To confirm this, we also performed cell count assays in two RMS cell lines (Figure S4C). We also assessed cell cycle arrest and apoptosis in RMS cells after KDM4B silencing. Although no apoptosis was detected (Figure S4D), ERMS cell lines showed marked cell cycle arrest after KDM4B silencing compared to ARMS cell lines (Figure 3C,D), indicating that KDM4B-silencing in ARMS cell lines results in a slowing of cell cycle progression rather than cell cycle arrest or apoptosis. Despite the reduction in cell growth, we did not see any increase in the expression of markers of cell differentiation in RMS cells (Figure S4E,F). Furthermore, siRNA silencing did not alter global levels of H3K9me in RMS cells (Figure S4G). 

### 3.4. KDM4B Regulates Transcription of G1/S and G2/M Checkpoint Genes

**Figure 4:**
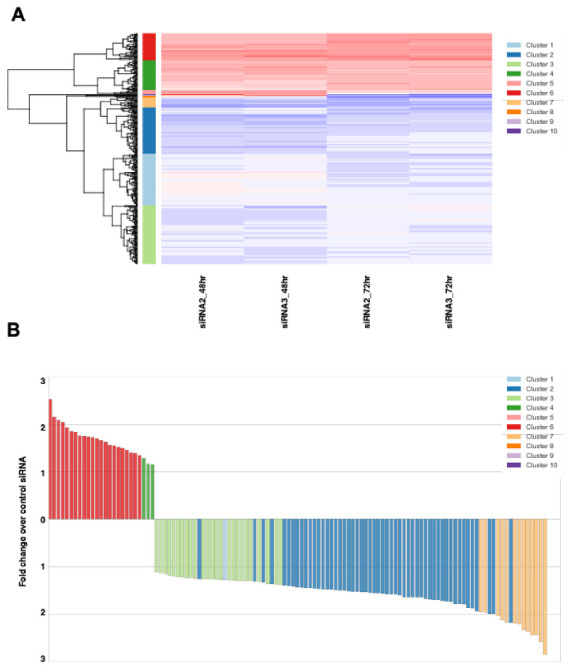

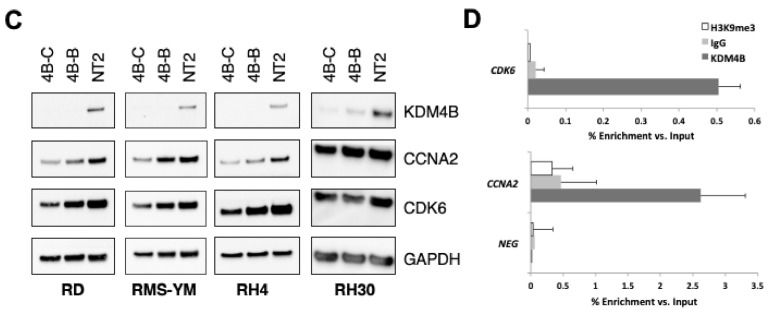
RNA-sequencing reveals a role for KDM4B in G1/S and G2/M cell cycle phases. (**A**) Heatmap showing changes in gene expression after KDM4B silencing relative to the Control siRNA at 48 and 72 h using two siRNAs. hierarchical clustering was carried out using the complete linkage method (**B**) Direction of change in expression of genes that change with both siRNAs at both time points after KDM4B silencing. Colors represent the cluster (from **A**) that each gene belongs to. (**C**) Western blots showing downregulation of G1/S checkpoint protein CDK6 and G2/M checkpoint protein CCNA2 after KDM4B silencing in two eRMS and two aRMS cell lines. (**D**) qChIP analysis in RH30 cells for KDM4B (dark grey bars), H3K9me3 (white bars) and IgG control (light grey bars) on the promoters of CDK6, CCNA2 and a negative control region. Uncropped Western Blot Images in Figure S11.

To determine which genes may be responsible for the phenotypic effects seen in ARMS cell lines, we investigated the transcriptional effect of silencing KDM4B in the ARMS line RH30 by RNA-sequencing at 48 and 72 h post-transfection. Gene Set Enrichment Analysis (GSEA) revealed both overlapping and distinct genes that were down-regulated at 48 h and 72 h post-transfection (Figure 4A, Figure S5A–D). Gene Ontology (GO) analysis of genes altered at each time-point showed that KDM4B predominantly regulates genes involved with cell cycle checkpoints throughout the cell cycle (Table S4), suggesting that regulation of cell cycle genes throughout the cell cycle contributes to the slowing of cell growth phenotype seen in ARMS cells. We then analyzed this data set for only those genes that were down-regulated or up-regulated at both 48 h and 72 h with two siRNAs (Figure 4B, Table S5), revealing four predominant clusters of genes. Genes within these clusters were more down-regulated (clusters 2, 3 and 7) or up-regulated over time (Figure S5E). Interestingly, we noted that >75% of genes were down-regulated after KDM4B silencing, suggesting that a predominantly activating H3K9me demethylase activity rather than a repressive H3K36me demethylase activity of KDM4B is likely responsible for regulating transcription of RMS genes. To determine which types of genes are under KDM4B control we then performed GO analysis on these clusters and found that genes that were consistently down-regulated were predominantly involved in cell cycle checkpoints, whereas up-regulated genes were involved in cell adhesion and angiogenesis (Table S6).

Of the genes that were down-regulated at either 48 h and/or 72 h after KDM4B RNAi we noted that a number of these are known to be involved in cell cycle checkpoint control at different phases of the cell cycle, including CDK6, a G1-S checkpoint gene, and CCNA2, a G2-M checkpoint gene. To confirm that KDM4B is regulating these genes in RMS we performed KDM4B silencing in four RMS cell lines and confirmed a reduction in CDK6 and Cyclin A2 (CCNA2) expression by Western blot in all cell lines (Figure 4C, Figure S6). In the ERMS line RD CDK6 and CCNA2 are expressed at lower levels than the RH30 cell line (Figure 4C). Thus, silencing of KDM4B in RD leads to markedly lower levels of CDK6 and CCNA2 compared to in RH30 cells and may explain the greater extent of cell cycle arrest seen in these cells. Consistent with regulating levels of these cell cycle genes we showed binding of KDM4B in the promoter regions of both CDK6 and CCNA2 by ChIP-PCR (Figure 4D). Furthermore, H3K9me3 was not detected at these loci, suggesting that KDM4B is directly controlling transcription of these genes (Figure 4D). 

### 3.5. Sustained KDM4B Silencing Leads to Resistance to Growth Suppression via Molecular Compensation by KDM4A

**Figure 5:**
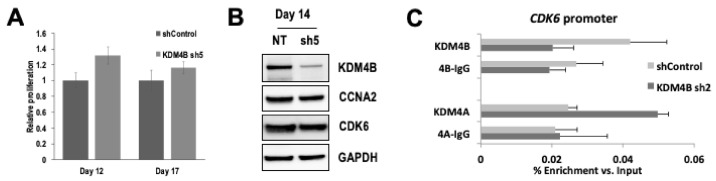
KDM4A compensates for KDM4B at cell cycle gene promoters after stable KDM4B silencing. (**A**) Long term stable silencing of KDM4B no longer results in a reduction in cell proliferation post-selection in RH30 cells. Cells were initially plated post-selection (day 5) and measured for rate of proliferation at day 12 and 17. Results are presented as mean ± SD. (**B**) Western blot showing no change in CDK6 or CCNA2 at day 14 post-transduction despite persistent KDM4B silencing. (**C**) KDM4A binds to the promoter of CDK6 in place of KDM4B after stable KDM4B silencing (Day 14 post-transduction). Uncropped Western Blot Images in Figure S11.

To determine the long-term effects of sustained KDM4B-silencing in RMS cells we tested 6 different shRNAs directed against KDM4B (Figure S7A). We measured cell proliferation in RMS cells post puromycin selection. Stable silencing of KDM4B showed a slight increase in proliferation in RH30 compared to control hairpin (Figure 5A, Day 12 *p* < 0.03, Day 17, *p* = NS, Figure S7B,C). Crucially, analysis of KDM4B-regulated cell cycle genes revealed that these were no longer down-regulated despite continued KDM4B silencing in these cells (Figure 5B,C). This was confirmed in RD cells where Stable silencing of KDM4B showed no difference in proliferation in RD cells post-selection (Figure S7B,D,E).

As functional redundancy has been postulated to exist between KDM4 sub-family members [2,3], we sought to determine whether other KDM4 family members might be up-regulated in cells after sustained KDM4B silencing. A slight increase in KDM4A expression was detected by Western blot, however no changes in KDM4C or KDM4D were detected in these cells (Figure S8A). However, when we repeated this analysis across multiple timepoints we saw no detectable changes in KDM4A expression (Figure S8B).

Despite a lack of upregulation in expression of other KDM4 family members, there remains the possibility of functional redundancy in regulating genomic targets of KDM4B in its absence. To test this hypothesis and determine whether the lack of reduction in expression of KDM4B-controlled cell cycle genes could be compensated for by the presence of KDM4A on these promoters we analyzed KDM4A binding on the promoter of CDK6 and CCNA2. In RH30 cells transduced with control shRNA, KDM4A does not bind to these promoters. However, after KDM4B silencing by shRNA, we observed an enrichment of KDM4A at these promoters, suggesting that KDM4A is now regulating expression of CDK6 (Figure 5C) and CCNA2 (Figure S9) in the absence of KDM4B. 

### 3.6. Dual Silencing of KDM4A and KDM4B Results in Apoptosis in RMS Cells

**Figure 6:**
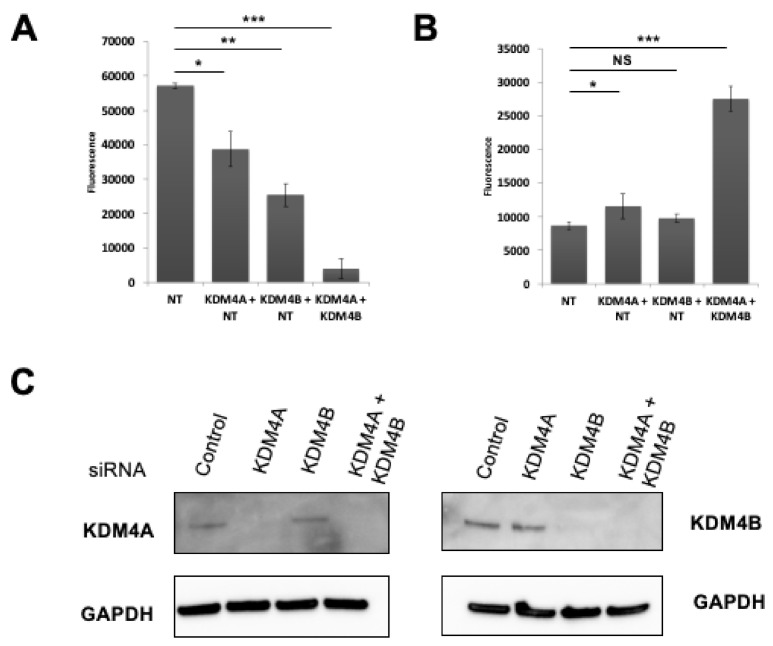
Dual up-front targeting of KDM4A and KDM4B results in apoptosis in RMS cells. (**A**) Cell proliferation after dual silencing as measured by CyQuant assay at 144 h post-transfection. Significance is indicated as *p* < 0.05 (*), *p* < 0.01 (**) and *p* < 0.001 (***), while NS indicates a non-significant *p*-value. (**B**) Combined silencing of KDM4A and KDM4B leads to apoptosis in RMS cells as measured by Caspase Glo. (**C**) Western blot showing knockdown of KDM4B and KDM4A in siRNA-transfected RH30 cells. Uncropped Western Blot Images in Figure S11.

As sustained silencing of KDM4B led to an increase in KDM4A at the CDK6 promoter we hypothesized that simultaneous targeting of both KDM4A and KDM4B may result in a stronger phenotype and thus mitigate the effects of stable silencing of KDM4B alone. siRNA-mediated dual targeting of KDM4A and KDM4B lead to a significantly greater decrease in RMS cell proliferation than with either KDM4A or KDM4B alone (Figure 6A,C). Furthermore, unlike targeting either KDM4A or KDM4B alone, dual targeting led to a highly significant increase in Caspase activity indicative of apoptosis in these cells (Figure 6B and Figure S10), suggesting that these two demethylase family members have distinct roles in RMS cell growth and that combined inhibition of both KDM4A and KDM4B is required in RMS to induce apoptosis and reduce the potential for resistance to KDM4 subfamily targeted therapies. 

## 4. Discussion

In this study, we demonstrate for the first time the role of KDM4B in controlling cell proliferation and cell cycle progression in RMS. Moreover, in this context, we also show the existence of functional redundancy between KDM4B and KDM4A in cell cycle regulation. Building on our previous study showing that HDMs are highly expressed in RMS compared to normal tissues, here we demonstrate that a number of HDMs affect RMS cell growth and show that silencing of KDM4 family members results in a reduction in cell proliferation in RMS cell lines including the H3K9me3 demethylase KDM4B. 

KDM4B has previously been shown to regulate *CDK6* in bladder and lung cancer cells [19]. We demonstrate that KDM4B also regulates *CDK6* in RMS and may therefore be a more widespread driver of proliferation in tumors. This phenotype is enhanced through the associated expression of several other cell cycle checkpoint genes known to be expressed at different phases of the cell cycle. Our RNA-sequencing data revealed that cell cycle genes, including *CDK6*, *CCNA2*, *SKP2* and *MYC* were all down regulated after KDM4B silencing. *SKP2* has been shown to be highly expressed in RMS and is a downstream target of the PAX3-FOXO1 fusion protein [20]. Thus, it is likely that KDM4B in conjunction with the fusion protein is responsible for the elevated expression of *SKP2* in RMS via H3K9me3 regulation. Interestingly, silencing of KDM4B in ERMS cell lines resulted in cell cycle arrest, compared to ARMS cell lines in which silencing of KDM4B results in a slowing down cell cycle progression. KDM4B has been shown to regulate different genes dependent on cell context (reviewed in [21]). As ARMS and ERMS cells have different pathologies and differing transcriptional profiles owing, at least in part to the presence of the fusion protein in ARMS [18] it is likely that KDM4B is regulating different genes in these two cell types, which may in turn account for the differences in cell cycle arrest. 

Here we show that KDM4B plays a role in the proliferative capacity of RMS cells. Indeed, in stem cells expression of KDM4 proteins is required for maintaining the undifferentiated, proliferative state and regulating differentiation [2,3,5,6]. RMS cells resemble undifferentiated skeletal muscle cells and thus present an undifferentiated phenotype that has been attributed to the overexpression of epigenetic proteins in these tumours [11,15,22,23], in line with data we present here for KDM4B. 

RNA-sequencing following KDM4B silencing also showed that the majority of genes that changed were down-regulated, consistent with transcriptional activation via H3K9me3 demethylation by KDM4B. However, around 25% of genes were upregulated after KDM4B silencing, suggesting that these genes are regulated by the H3K36me3, a hallmark of active transcription, implicating a demethylating, repressive role of KDM4B on these genes. Mutations in histone H3 lysine 36 to methionine (H3K36M) have been shown to prevent differentiation in mesenchymal progenitor cells (MPCs) and have been linked to sarcomagenesis in mouse models [24]. Silencing of H3K36 methyltransferases was shown to phenocopy such mutations leading to impaired differentiation in MPCs. This suggests that the high expression of H3K36 demethylating enzymes found in RMS (including KDM2 and KDM4 family members) are worthy of future investigation for their likely role in keeping RMS in a less differentiated and proliferative state. The H3K9 histone methyltransferase G9a has previously been shown to regulate tumor growth in ARMS by regulating the PTEN-AKT-RAC1 axis in these cells [25]. Although we do not see consistent downregulation of either PTEN or RAC1 in our siRNA silencing data, we do consistently see downregulation of AKT3 at both timepoints with both siRNAs, in line with the idea that KDM4B and G9a may be regulating AKT3 in an opposing fashion in these cells.

Although short-term silencing of KDM4B by siRNA in RMS cells led to a reduction in proliferation, long-term stable silencing by shRNA did not. This is in contrast to reports in neuroblastoma, where stable silencing of KDM4B led to a reduction in cell proliferation and a concomitant induction of differentiation in these cells [26]. In RMS this is likely due to functional redundancy by KDM4A, which we show is able to molecularly compensate for the lack of KDM4B by binding to the promoter of KDM4B-regulated cell cycle check point genes to keep H3K9me3 demethylated. This suggests that, at least in the case of RMS, single targeting of KDM4B alone as a therapeutic strategy may not be effective in these cells. Indeed, consistent with this, Yang et al. showed that treatment of MYC-positive neuroblastoma cells with ciclopirox, an antifungal agent that also acts as a pan-demethylase inhibitor, resulted in a reduction in NB cell viability [27].

Over the past few decades drug discovery efforts have commonly sought a single molecule that selectively targets a single protein. However, functional redundancy between protein family members and/or signaling pathways have been shown to be potential mechanisms of resistance to therapies in the treatment of cancer [28,29], and many of the most efficacious cancer drugs have multiple protein targets. However, broad-range poly-pharmacology often leads to toxic/harmful side and/or off-target effects in patients (reviewed in [30]). Recent advances in epigenetic therapies appear to be bridging the gap between the traditional single target paradigm for drug discovery and the advantages of poly-pharmacology for therapeutic intervention [31]. Unlike kinases, distinct sub-families of epigenetic modulators have specificities for differing substrates resulting in distinct molecular epigenetic consequences, and thus presents an opportunity for sub-family selectivity in drug design. For example, specific HDM sub-families have specificity for differing residues on histone tails, such that multiple histone demethylase sub-family members are able to demethylate the same specific residue on a given histone tail, although they may be guided to different genomic loci. This also suggests that functional redundancy may exist between sub-family members. Indeed, mouse knockouts of Kdm5A have a relatively mild phenotype indicative of functional redundancy [32]. Furthermore, combined inhibition of all four KDM5 sub-family members has recently been shown to be effective against treatment-resistant cancer cells [33]. Thus, effective utilization of selective poly-pharmacology could prevent the emergence of resistance. In our RMS model, the emergence of functional redundancy between KDM4A and KDM4B on the promoters of cell cycle genes after stable KDM4B silencing highlights the need for multi-KDM4 targeting in these tumors in order to prevent resistance. Furthermore, the emergence of an apoptotic phenotype with the combined silencing of KDM4B and KDM4A in RMS cells further highlights the advantage of dual targeting of these two KDM4 family members for the treatment of RMS. 

There has been a recent drive to find KDM4 inhibitors, although very few of these studies have been able to derive selective compounds without poly-pharmacology with other KDM families, largely due to the fact that they are either cofactor mimics or substrate competitive, both of which are often shared across histone demethylase families. However, some selectivity has been achieved using cyclic peptides, which even allow for some selectivity over subfamily members [34]. Based on the data we present we believe that it will be necessary to inhibit both KDM4A and KDM4B to achieve clinically significant results but, as there are currently no selective KDM4 family inhibitors, it is not possible to recommend a specific inhibitor that could be developed for use in RMS. 

Overall, the data we have presented highlights the importance of understanding the underlying functional redundancy that may exist between histone demethylase family members in order to effectively target these epigenetic proteins for the effective treatment of cancer. Our discovery that KDM4A is able to compensate for down-regulated KDM4B by assuming the latter’s H3K9me3 demethylating role to drive transcription of KDM4B-regulated genes highlights the potential for sub-family targeting of these proteins rather than striving for single-target therapies. KDM4A itself has been associated with mechanisms that may lead to resistance [35], thus it is desirable to target both KDM4A and KDM4B in order to reduce the potential for resistance to single-target agents.

Furthermore, our data show that effectively targeting both KDM4A and KDM4B together leads to apoptosis in RMS cells and thus suggests that effective dual targeting of these proteins could lead to a more effective therapy, whilst maintaining the benefits of more targeted therapies with reduced toxicities. 

## 5. Conclusions

A number of histone demethylases are highly expressed in rhabdomyosarcoma and impact on cell proliferation, including KDM4B. We determine that KDM4B regulates the expression of cell cycle genes in rhabdomyosarcoma. However, after clonal selection of KDM4B-silenced cells, KDM4A is able to functionally compensate for the regulatory effects of KDM4B on the promoters of the cell cycle genes CDK4 and CCNA2. We conclude that, in order to mitigate for this functional redundancy and enhance therapeutic potential for rhabdomyosarcoma patients, a dual targeting approach to pharmacological inhibition of these histone modifying enzymes will be necessary.

## Data Availability

Raw data for the ITCC/CIT collection analysed in this study are available in the ArrayExpress database (accession ID E-TABM-1202).

## Supplementary Materials

The following are available online at https://www.mdpi.com/article/10.3390/cancers13071734/s1, Figure S1. Z-scores of all siRNAs from HDM screen in RMS cell lines, Figure S2. (A) Expression of KDM4B in RMS patient samples from Affymetrix gene expression profiling data. One-way ANOVA conducted for patient samples versus skeletal muscle. (B) KDM4B is more highly expressed in RMS patient samples than skeletal muscle (*p* < 0.0001) (C) qRT-PCR expression of KDM4B in normal tissue samples, Figure S3. Antibodies used for TMA analysis, Figure S4. Confirmation of KDM4B expression and silencing in RMS cell lines, Figure S5. RNA-sequencing after KDM4B silencing in RH30 cells, Figure S6. Densitometry of Westerns showing a decrease in CCNA2 and CDK6 in RMS cell lines, Figure S7. shRNA silencing of KDM4B, Figure S8. Expression of KDM4 Family members after KDM4B shRNA silencing, Figure S9. KDM4A binds to the promoter of CCNA2 in place of KDM4B after stable KDM4B silencing, Figure S10. siRNA silencing of KDM4A in KDM4B shRNA-silenced cells, Figure S11. Full length blots. Table S1. Results of siRNA screen in RD and RH30 after normalisation to Control siRNA and *Z*-scores of each individual siRNA compared to control, Table S2. KDM4B protein staining in RMS patient samples by histology, Table S3. KDM4B protein staining in RMS patient samples by clinical features, Table S4, Correlation between KDM4B and Ki67 protein staining in RMS patient samples, Table S4. GO analysis on genes significantly changed after KDM4B RNAi, Table S5. Genes significantly altered at 48 h and 72 h after KDM4B RNAi, Table S6. GO analysis of gene clusters, Table S7. Antibodies used in this study.
